# ADP/ATP translocase 1 protects against an α-synuclein-associated neuronal cell damage in Parkinson’s disease model

**DOI:** 10.1186/s13578-021-00645-x

**Published:** 2021-07-10

**Authors:** Wenyong Ding, Minghua Qi, Li Ma, Xuefei Xu, Yingfei Chen, Wenli Zhang

**Affiliations:** 1 Biochemistry and Molecular Biology Department of College of Basic Medical Sciences, Dalian Medical University, Dalian, 116044 China; 2 Department of Epidemiology, Dalian Medical University, Dalian, 116044 China; 3 Grade 2020, Capital Medical University, Beijing, 100069 China

**Keywords:** ADP/ATP translocase 1, Parkinson’s disease, PD, ANT1, α-synuclein

## Abstract

**Background:**

ADP/ATP translocase 1 (ANT1) is involved in the exchange of cytosolic ADP and mitochondrial ATP, and its defection plays an important role in mitochondrial pathogenesis. To reveal an etiological implication of ANT1 for Parkinson’s disease (PD), a neurodegenerative disorder, a mouse model treated with 1-Methyl-4-phenyl-1,2,3,6-tetrahydropyridine and neuroblastoma cell model induced by 1-methyl-4-pehny1-pyridine were utilized in this study.

**Results:**

The tissue-specific abundance in ANT1 in mouse brains was accessed using the analysis of Western blot and immunohistochemistry. Down-regulated soluble ANT1 was found to be correlated with PD, and ANT1 was associated with PD pathogenesis via forming protein aggregates with α-synuclein. This finding was confirmed at cellular level using neuroblastoma cell models. ANT1 supplement in neuronal cells revealed the protective roles of ANT1 against cytotoxicity caused by MPP^+^. Protein interaction assay, coupled with the analysis of LC-MS/MS, silver-stained SDS-PAGE and Western blot against anti-ANT1 antibody respectively, illustrated the interaction of ANT1 with α-synuclein using the expressed α-synuclein as a bite. Additionally, a significant increasing ROSs was detected in the MPP^+^-treated cells.

**Conclusions:**

This study indicated that ANT1 was a potentially causative factor of PD, and led to neuropathogenic injury via promoting the formation of protein aggregates with α-synuclein. This investigation potentially promotes an innovative understanding of ANT1 on the etiology of PD and provides valuable information on developing potential drug targets in PD treatment or reliable biomarkers in PD prognostication.

## Introduction

Parkinson’s disease (PD) is characterized as a slowly progressive neurodegenerative disorder that results from a combination of multiple genetic and environmental risk factors [1–3]. This disease affects approximately 1% of the population above 60 years old of age. It is estimated that overall incidence of disease ranges from 10 to 18 per 1,00,000 person-years globally, and a continuous rise in the number of PD patients is expected in the future [4]. This trend has profound public health implications for a rising life expectancy among aging population, and results in extensive socioeconomic burden of PD worldwide [5, 6].

The patients with PD decrease their life span, reduce their life quality and suffer a lot from the disease. The parkinsonian symptoms clinically include motor features, such as rest tremor, muscular rigidity and bradykinesia, and non-motor symptoms containing autonomic dysfunction, cognitive impairment, sleep disorders, psychiatric symptoms, dementia, etc. Since non-motor symptom appears at onset, the patients with PD may develop into classical motor features after long-term disease duration as the disease progresses [1, 4, 5, 7, 8].

Nowadays, PD is characterized by the loss of dopaminergic neurons within the *substantia nigra pars compacta* (*SNpc*) which contributes to the motor impairment of PD. However, the recent growing evidences illustrates that pathologic area of PD is distributed outside the nigrostriatal pathway and tends to appear prior to the onset of overt motor symptoms. Meanwhile, as aggregates of abnormally folded proteins has been identified to be a common pathogenesis of neurodegeneration, the accumulation of intracellular protein inclusions which composes primarily of α-synuclein is found to be an important biological hallmark of PD. Till now, α-synuclein is recognized as a major component to form a variety of different aggregates with thin thread-like or small dot-like structures in the brain with neurodegenerative disease [1, 9].

Although several great advances in parkinsonian hallmark have been discovered, the pathogenesis and etiology of PD still are poorly understood [10]. Furthermore, there is no effective pharmacological treatment that fully cures it and preventive strategy that palliates the disease progress. The present therapeutic treatments only alleviate the symptoms associated with PD. Therefore, there is a strong need to understand the molecular mechanism of PD pathogenesis that will help researchers to identify novel therapeutic targets leading to new treatment strategies.

ADP/ATP translocase 1 (ANT1), participating in the exchange of cytosolic adenosine diphosphate (ADP) and mitochondrial adenosine triphosphate (ATP) across the inner mitochondrial membrane, is the main mitochondrial ADP/ATP exchanger with major metabolic implications [11]. Nowadays, ANT1 have been intensively studied in myocardial contractile system [12–15] and tumor [16–18]. However, little is known about ANT1 contributing to PD. To investigate the effect of ANT1 on the pathology of PD, a neurotoxin-based animal model induced by 1-methyl-4-phenyl-1,2,3,6-tetrahydropyridine (MPTP) and cellular model treated by 1-methyl-4-phenylpyridinium ion (MPP^+^), were utilized in this study. MPTP, a lipophilic molecule, can easily cross the blood brain barrier and be oxidized into 1-methyl-4-phenylpyridinium ion (MPP^+^), a potent dopaminergic neurotoxin by the monoamine oxidase B in astrocytes. MPP^+^ readily enters the nigrostriatal neurons via dopamine transporters and induces progressive loss of dopaminergic neurons in the *SNpc* contributing to striatal dopamine depletion further causing a parkinsonian syndrome [19, 20]. Till now, the MPTP-treated PD animal models have been commonly used to unravel various pathological events and explore therapeutic mechanisms due to the similar clinical symptoms in animal to those in patients with PD, reliable and reproducible lesions in the nigrostriatal dopaminergic pathway, and less requirements for experimental technology [19, 21, 22]. In order to create a cellular model that mimicked PD, we exposed the neuroblastoma SH-SY5Y cells to MPP^+^ in this study [23].

In this study, we aimed at elucidating an association of ANT1 with the neuropathology of PD via the MPTP-treated mouse models and cellular model induced by MPP^+^, further to investigate the molecular mechanism of PD etiology and pathogenesis.

## Materials and methods

### Ethical approval

This study was carried out in accordance with the principles of the Basel Declaration and recommendations of Dalian Medical University for laboratory animals. The protocol was approved by the Animal Ethics Committee of Dalian Medical University.

### Construction of the MPTP-treated PD mouse model

Fifty-two C57BL/6 male mice, weighting 20–25 g and 8–10-week-old, were randomly divided into two groups: control (n  =  26) and MPTP (n  =  26). The intraperitoneal injection with MPTP (25 mg/kg or 10 ml/kg, dissolved in physiological saline) was preformed onto mice ten times at intervals of 3.5 days in the MPTP group. Meanwhile, the mice were treated with the same volume of physiological saline (10 ml/kg) via intraperitoneal injection in control group. Locomotor activity was examined and the parkinsonian biological markers including 5-HT and DA were detected by RP-HPLC, and TH was tested by immunohistochemistry (IHC) as described previously [24].

### Cell culture, treatment and cell viability assay

Neuroblastoma SH-SY5Y cells were routinely grown in Dulbecco’s modified Eagle’s medium/F12 nutrient mixture (DMEM:F12) supplemented with 10% (v/v) heat-inactivated fetal bovine serum (FBS) and 100 units/ml of penicillin/streptomycin. Cells were cultured at 37 °C under a humidified atmospheric condition containing 5% carbon dioxide. To investigate PD-like neurotoxicity induced by MPP^+^, SH-SY5Y cells were normally grown for 24 h followed by incubation with MPP^+^ at various concentrations for another 24 h. The morphology of cells was examined under an inverted microscope. The optimum MPP^+^ concentration was determined by plotting cell viability against MPP^+^ contents. Cell viability was evaluated by MTT assay in 96-well plates. After treatment with MPP^+^, the SH-SY5Y cells were incubated with 100 μl of MTT solution (0.5 mg/mL in PBS) at 37 °C for another 4 h. Then, DMSO was added into cells in order to dissolve formazan crystals, and the absorbance at 490 nm was read on a microplate ELISA reader (Thermo Scientific). All experiments were performed independently in triplicate.

### Preparation of protein lysates

For mice, on the 7th day after the last MPTP injection, the mice in each group were sacrificed by cervical dislocation. The mouse brains were separated and washed with ice-cold 0.9% physiological saline. The different specialized structures of mouse brain, including striatum, midbrain, cerebellum, cortex, hippocampus, brain stem were dissected carefully, and homogenized in ice cold RIPA lysis buffer [50 mmol/L Tris (pH7.4) containing 150 mmol/L NaCl, 1% Triton X-100, 1% sodium deoxycholate, 0.1% SDS and 1 mmol/L PMSF] followed by clearance at 14,000 rpm for 30 min twice. For cells, the cultured SH-SY5Y cells in a 10 cm dish were digested with trypsin, and collected by centrifugation at 1000 rpm for 5 min. Then, the SH-SY5Y cells were broken by ultrasonication (5S on, 3S off) followed by removal of insoluble fragments through centrifugation at 14,000 rpm for 30 min twice. Subsequently, the protein concentrations of the supernatants were determined using a BCA kit (keyGEN BioTECH, China). Thus, the protein lysates were obtained and frozen at − 80 °C for a further investigation.

### Processing of fixed brain tissues

On the 7th day after the last MPTP injection, the mice were anesthetized by inhalation of diethyl ether, and mechanically fixed to expose the heart completely via opening the chest. The mice were intracardially perfused with 0.9% physiological saline and 4% paraformaldehyde in phosphate-buffered saline (PBS; 50 mmol/L of NaH_2_PO_4_; 5 mmol/L of KCl; 1.5 mmol/L of MgCl_2_; and 80.1 mmol/L of NaCl; pH 7.4) for 30 min respectively. On the one hand, the different specialized structures including striatum, midbrain, cerebellum, cortex, hippocampus, brain stem were dissected carefully, post-fixed in the above fixative, and stored at 4 °C for IHC examination. On the other hand, the whole mouse brains were saturated in 15% picric acid in PBS followed by storage in 20% sucrose in PBS at 4 °C for immunofluorescence staining.

### Reversed phase high performance liquid chromatography (RP-HPLC) analysis of DA and 5-HT content

The equal volume of cold acetone was added for removing high-abundance proteins from the striatal homogenate of mice and cell lysate of neuroblastoma SH-SY5Y away. The level of dopamine (DA) and 5-hydroxytryptamine (5-HT) in SH-SY5Y cells and striatum of mouse brains were measured by performing RP-HPLC respectively. The samples were injected into HPLC analysis, and eluted through a C18 column (3.9 mm  ×  150 mm, Thermo Fisher Scientific, Waltham, MA, USA). The mobile phase was a mixture of acetic acid buffer (pH 3.5, containing 12 mmol/L acetic acid, 0.26 mmol/L EDTA disodium): methanol  =  86:14; the flow rate was constant at 0.5 ml/min over the course of HPLC separation, and the eluted compounds were detected by monitoring the absorbance at the wavelength of 280 nm.

### Western blot analysis (WB)

Eighty microgram of soluble proteins for each different specialized structures and neuroblastoma SH-SY5Y cells was separated by running a 15% SDS-acrylamide gel electrophoresis (SDS-PAGE) in a vertical electrophoresis apparatus and transferred to a PVDF membrane in blotting buffer (20 mmol/L Tris-base, 150 mmol/L glycine and 20% methanol) to make protein accessible to monoclonal antibody detection using anti-β-actin antibody (Abcam), anti-ADP/ATP translocase 1 antibody (anti-ANT1, Abcam) and anti-GAPDH antibody (Abcam). The protein bands were visualized by an enhanced chemiluminescent (ECL) system (GE Healthcare Bio-Sciences Corp.).

### Immunohistochemistry (IHC)

The different fixed specialized structures were routinely processed for embedding in a paraffin block followed by dehydration using a gradient concentration of alcohol (70, 80, 90, 95, 100% respectively). Then, the samples were diaphanized by immersion in xylol, and embedded in paraffin. The paraffin-embedded sections were sliced to 10 μm thickness and mounted on glass slides for IHC analysis using the primary antibody of anti-ANT1 and anti-TH (tyrosine hydroxylase, Santa Cruz Biotechnology, Santa Cruz, CA). Diaminobenzidine was used for visualization of immunolabeling.

### Detection and quantification of intracellular ROSs

Measurement of the intracellular ROSs was performed using the fluorescent probe 2′,7′-dichlorofluorescin diacetate (DCFH-DA; Sigma-Aldrich), which can be oxidized to the highly fluorescent dichlorofluorscein (DCF). Cells were seeded and cultured onto black 96-well plates with a clear bottom. After exposure to MPP^+^ for another 24 h, SH-SY5Y cells were incubated with DCFH-DA (10 μmol/L) for 30 min. After washing with PBS twice, the fluorescence intensity was determined using a fluorescence microplate reader (Beckman) with an excitation wavelength of 485 nm and an emission wavelength of 535 nm.

### Immunofluorescence staining

After the whole mouse brain was frozen in Milli-Q water in freezing microtome/cryostat (− 40 °C), six micron-thick sections were cut and mounted on glass slides for immunofluorescence analyses using the monoclonal antibodies including anti-ANT1 and anti-α-synuclein as the probes. SH-SY5Y cells were seeded on poly-L-lysine-coated coverslips in six-wells plates for 12 h. Then, the cells were fixed with 4% paraformaldehyde followed by permeabilization with 0.5% Triton X-100. After blocked with 1% normal serum in PBS for 30 min at room temperature, the SH-SY5Y cells were incubated with monoclonal antibodies, including anti-ANT1 and anti-α-synuclein respectively at 4 °C overnight. The secondary antibodies conjugated to rhodamine/FITC were used in this study. All samples were counterstained with 4′,6-diamidino-2-phenylindole (DAPI) (Sigma-Aldrich, St. Louis, MO, USA). The images were acquired with an Olympus inverted fluorescence microscope (Olympus Corporation of the Americas, Center Valley, PA, USA).

### The construction of pcDNA3.1-*hsa-ANT1*

The *hsa-ANT1* gene (GenBank: BC022032.2) was amplified from cDNA of homo sapient using the forward primer of 5′ ATAAGCTTCGAGCTGTCACCATGGGTGATC 3′ (underlined sequence was HindIII site) and the reverse primer of 5′ TCCTCGAGTTAGACATATTTTTTGATCTCATC 3′ (underlined sequence was XhoI site). The purified *hsa*-*ANT1* fragment was cloned into the pJET-T to generate pJET-*hsa*-*ANT1.* Then the sequence-confirmed *hsa*-*ANT1* from the plasmid of pJET-*hsa*-*ANT1* was cloned into pcDNA3.1 to yield pcDNA3.1-*hsa*-*ANT1* for transfecting neuroblastoma SH-SY5Y Cells.

### Transfection

Neuroblastoma SH-SY5Y Cells were seeded in flat-bottomed 6-well plates and exposed to 1.2 mM MPP^+^. Then, the MPP^+^-treated cells were cultured for transfection until they reached a confluence of 70–90%. And the transfection was conducted using lipofectamine 2000 (Thermo Fisher Scientific, Waltham, USA). Prior to transfection, the plasmid of pcDNA3.1-*hsa*-*ANT1* was mixed directly with lipofectamine 2000 (m:v  =  1:2–3) in DMEM. Then, the mixture was added to the cells. Transfection efficiency, cell apoptosis and parkinsonian parameters were monitored at 48 h post-transfection.

### Flow cytometric analysis of cell apoptosis

Cell apoptosis was analyzed using flow cytometry and Annexin V-FITC Apoptosis Detection Kit (Jiangsu Kaiji Biotech, Jiangsu, China). SH-SY5Y cells were stained with dual staining Annexin V-fluorescein isothiocyante (FITC)-propidium iodide (PI) at room temperature in the dark for 15 min. Then, the stained cells were measured by flow cytometry (Beckman-CytoFLEX Coulter, CA, USA). The data were analyzed by FlowJo (BD Biosciences, NJ, USA), and the apoptotic cells were counted and presented as a percentage of the total cell counts. The results were conducted in triplicate.

### Expression, purification and detection of α-synuclein expressed in *Escherichia coli* BL21 (DE3)

α-*synuclein* gene was chemically synthesized and sequenced followed by ligation with pCold II to yield the expression vector of pCold II-α-*synuclein* in Takara company. The pCold II-α-*synuclein* was transformed into *E. coli* BL21 (DE3) cells for over-expression of the α-synuclein fusion protein. A single colony of *E. coli* BL21 (DE3) carrying pCold II-α-*synuclein* was grown in 5 ml of LB medium overnight, and the resulting culture was used to inoculate 200 ml of LB medium. When the optical density of the culture was 0.5 at 600 nm, 0.6 mmol/L isopropyl-D-thiogalactopyranoside (IPTG) was added to induce α-synuclein expression for another 20 h at 16 °C. The cells harboring pCold II-α-*synuclein* were lysed by sonication (30 s pulse with 30 s interval for 20cycles) in pre-chilled lysis buffer [pH8.0, 50 mmol/L NaH_2_PO_4_, 300 mmol/L NaCl, 20 mmol/L imidazole, 1 mmol/L phenylmethyl sulphonyl fluoride (PMSF)]. The cytoplasmic fractions were applied to a pre-equilibrated Ni–NTA column (Sigma, St. Louis, MO, USA) of 1.0 mL column volume after they were clarified by centrifugation at 27,000*g* at 4 °C for 40 min. The purification was performed according to the manufacturer’s instruction. The column was washed with 20 ml wash buffer (pH8.0, 50 mmol/L NaH_2_PO_4_, 300 mmol/L NaCl, 40 mmol/L imidazole, 1 mmol/L PMSF) to remove unbound protein, and eluted with 10 ml elute buffer (pH8.0, 50 mmol/L NaH_2_PO_4_, 300 mmol/L NaCl, 250 mmol/L imidazole, 1 mmol/L PMSF). The eluted fractions were collected, and their protein concentration was determined using a BCA kit. α-synuclein in the eluted fractions was verified by running a 15% SDS-PAGE followed by Western blot analysis. The probing of the membrane with antibody of (anti)-polyhistidine monoclonal HIS-1 (Sigma, St. Louis, MO, USA) was conducted manually, and the colorimetric detection of protein bands was developed by ECL solution [25].

### Identification of putative α-synuclein interaction partners

Pull-down assay for α-synuclein interaction partners was performed using Ni–NTA magnetic agarose beads (Qiagen, Hong Kong, PRC). The purified His_6_-tagged α-synuclein (bait, 8 μg) was allowed to bind to the Ni-NTA magnetic agarose bead suspension (40 μl) in 500 μl pull-down reactions under gentle rotation at 4 °C for 2 h. Then, the supernatants were removed using a magnetic MagRack6™ (Qiagen, Hong Kong, PRC) whereas the magnetic beads bound with α-synuclein protein were remained in a microcentrifuge tube. Five hundred microliters of whole-cell lysate of mouse brains with protease inhibitor cocktail (Sigma, St. Louis, MO, USA) (prey) was added to the α-synuclein-bound Ni-NTA beads and incubated at 4 °C for 2 h with gentle rotation. After incubation, unbound proteins were removed by washing using wash buffer (pH8.0, 50 mmol/L NaH_2_PO_4_, 300 mmol/L NaCl, 40 mmol/L imidazole, 1 mmol/L PMSF). The beads were then suspended in 50 μl elute buffer (pH8.0, 50 mmol/L NaH_2_PO_4_, 300 mmol/L NaCl, 250 mmol/L imidazole, 1 mmol/L PMSF), and the eluted fractions were collected. The bound proteins were run on a 15% SDS-PAGE gel stained with a silver staining kit (Sigma, St. Louis, MO, USA), and analyzed by Western blot analysis coupled with a 15% SDS-PAGE separation. The probing of the membrane with (anti)-ANT1 monoclonal antibody was conducted manually, and the colorimetric detection of protein bands was developed by ECL solution.

### LC–MS/MS

Silver-stained gel lane was excised into small pieces followed by decolorization in 400 μl mixed solution containing 100 mmol/L Na_2_S_2_O_3_: 300 mm K_3_Fe(CN)_6_ (1:1). The reduction of the decolorized gel was performed in 100 mmol/L NH_4_HCO_3_ solution, and the digestion of the gel was conducted in 50 mmol/L NH_4_HCO_3_ solution containing 12.5 ng/μl of sequencing-grade trypsin for 20 h at 37 °C according to a modified in-gel trypsin digestion procedure. Then, the trypsin-digested peptide fragments were desalted using C18 StageTip column followed by lyophilization. The lyophilized peptide fragments were dissolved in 0.1% formic acid for subsequent liquid chromatography-tandem mass spectrometry (LC-MS/MS) analysis.

The LC-MS/MS analysis was performed using shotgun separation of peptide mixtures on a reversed phase C18 column (75 µm*150 mm, 3 µm, C18, Thermo Scientific, Waltham, USA) by EasynLC1200 chromatographic system (Thermo Scientific, Waltham, USA). Optimum separation was achieved with a binary mobile phase at a flow rate of 300 nl/min. Solvent A: 0.1% formic acid aqueous solution; solvent B: 0.1% formic acid, 95% acetonitrile and water. The gradient elution program was: 0–3 min, ramping to 7% from 2% of solution B; 3–48 min, ramping to 35% from 7% of solution B; 48–53 min, ramping to 90% from 35% of solution B; 53–60 min, 90% of solution B. The MS analysis was conducted by Q-Exactive Plus mass spectrometer in 60 min. The Q-Exactive Plus MS analysis was set to data-dependent acquisition, and the MS instrument was operated in the positive mode. Working conditions of MS were as follows: mass range 300–1800 m/z at 70,000 at m/z200 resolution for triggering MS/MS events; automated gain control (AGC) at 1e6; maximum IT at 50 ms. The data of tandem MS was acquired under the following conditions: 20 ions with the highest intensity triggered by each full-scan of MS; the resolution of the tandem mass spectrum: 17,500 at m/z200; AGC: 1e5; maximum IT: 50 ms; MS2 Activation Type: HCD; Isolation window: 2.0Th, Normalized collision energy: 27. The MS data retrieval was performed by MaxQuant1.6.1.0 software against Uniprot Mus musculus Database.

### Image quantification and statistical analyses

Quantification of the protein bands was conducted using Image J (version 1.42). The color images were converted to gray-level intensity for quantification. Slight variations in background staining were corrected by subtracting background density. Digital analysis on the immunohistochemistry images was performed using Image-Pro Plus software (version 6.0).

Statistical analyses were conducted using GraphPad Prism 6 and SPSS (version 19). The statistical difference between the MPTP-treated group and the control group was evaluated using a two-tailed equal variance Student’s *t *test. WB and IHC data were analyzed in triplicate. A threshold of *P* values  <  0.05 was considered statistically significant.

## Results

### Identification of essential parkinsonian symptoms for the constructed MPTP-treated mice

MPTP, which can reproduce the similar essential parkinsonian symptoms in mice to the ones in patients with PD, was utilized for preparing animal model of PD in this study. A successful mouse model was supported by detecting the reduction in the representative biochemical markers of PD including dopamine (DA), 5-hydroxytryptamine (5-HT) and tyrosine hydroxylase (TH), and examining motor dysfunction via the vertical grid test which assesses the fore-paw strength and horizontal grid test which evaluates the fore-paw faults.

**Fig. 1:**
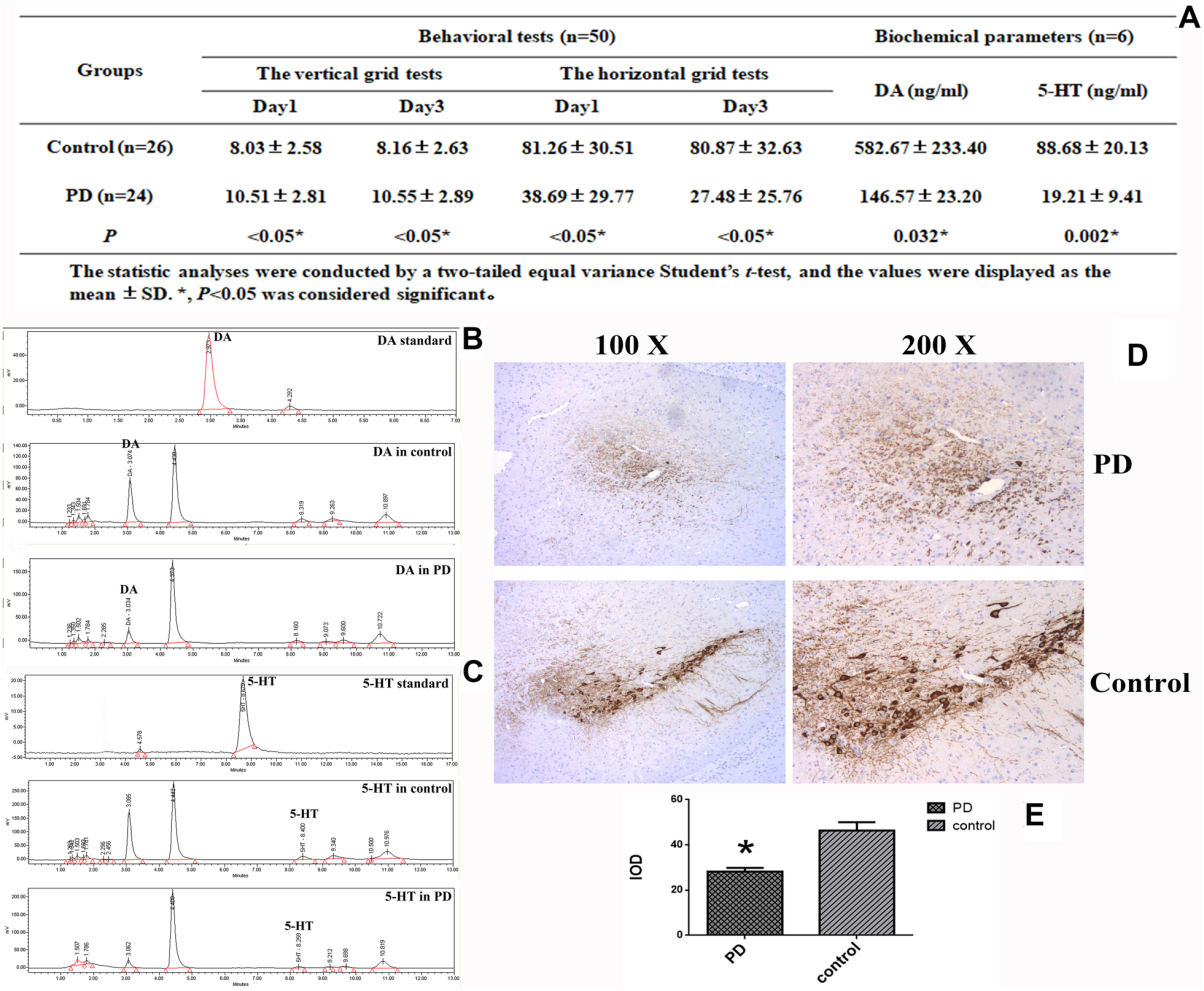
Verification of the essential parkinsonian symptoms in the MPTP-treated group. **a** Verification of the mice treated by MPTP based on the test of behavioral activity and the detection of the representative biochemical markers (two were dead in the MPTP group). **b** Representative HPLC analysis on striatal DA and its standard. **c** Representative HPLC analysis on striatal 5-HT and its standard. **d** Representative micrographs of IHC staining for TH. **e** Statistical analysis of IHC staining for TH between the MPTP-treated group and control group. The statistic analyses were conducted by a two-tailed equal variance Student’s *t *test based on the IHC micrographs taken at the magnification of 200; bars represented the mean  ±  SEM; *P  <  0.05

Typical parkinsonian symptoms are motor dysfunction. Thereby, in order to assess whether the MPTP administrated mice had motor impairment, a locomotor activity was evaluated between the healthy and MPTP-treated group. Figure 1A shows that the impairment of motor function is found in the MPTP-treated group. The comparison analysis on the horizontal grid test showed a dramatic reduction in hanging duration in the MPTP-treated group relative to the control group (Fig. 1A). And the vertical grid test displayed a significant increase in time for the mice to climb down the pole from the top in the MPTP-treated group compared to the control group (Fig. 1A).

In support of behavioral tests, an analysis on the representative parkinsonian parameters including DA and 5-HT in striatum examined by RP-HPLC, and TH in *SNpc* evaluated by immunohistochemistry was conducted. The MPTP-treated mice displayed a significant reduction in striatal DA (Fig. 1A, B) and striatal 5-HT (Fig. 1B, C) compared to the mice in the control group. A strong immunoreactivity against the monoclonal anti-TH antibody was found in *SNpc* in the control group as shown in Fig. 1D, and the quantification of IHC micrographs revealed that a massive loss of TH with statistical significance was occurred in the MPTP-treated group relative to the control (Fig. 1E). Thus, a dramatic decrease in striatal DA, 5-HT and mesencephalic TH was found in the MPTP-treated group.

As a result, the representative parkinsonian symptoms including the impaired motor functions, a reduction in DA and 5-HT in striatum, a decrease in TH in *SNpc*, were found in the MPTP-treated group. Therefore, these evidences demonstrate that PD mouse model constructed by intraperitoneal MPTP administration displays the same characteristic parkinsonian symptoms as patients with PD.

### Reduced soluble ANT1 was associated with PD

**Fig. 2:**
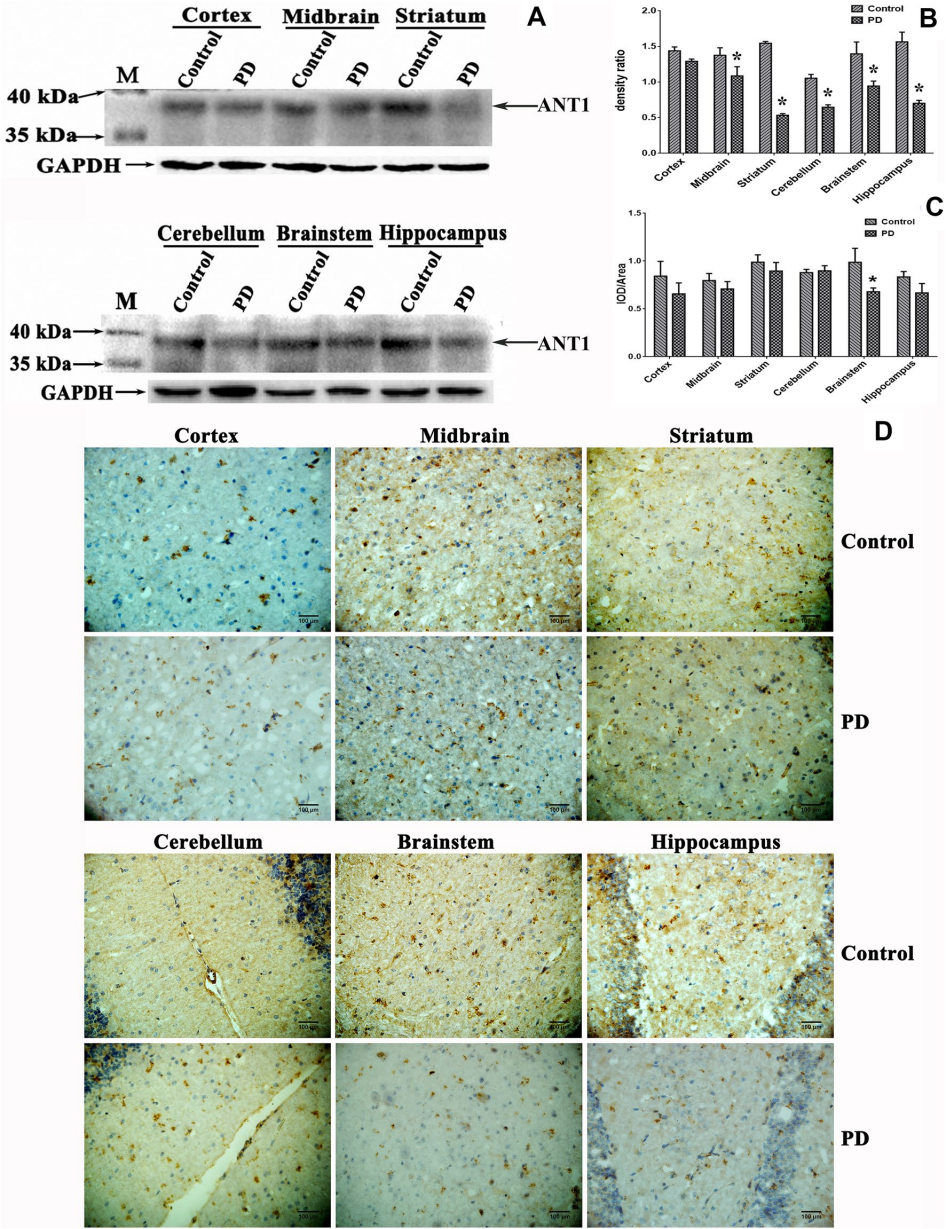
Association of ANT1 with the risk of PD. **a** Analysis of ANT1 abundance in various structures of the mouse brains detected by Western blot analysis. Cells lysates containing 80 μg soluble proteins were subjected to Western blot analysis coupled with 15% SDS-PAGE separation. The membrane was probed with monoclonal anti-ANT1 antibody and visualized using a ECL kit. M, Page Ruler prestained protein ladder (Fermentas). **b** Densitometric analysis of ANT1 normalized to the level of GAPDH. The analyses were conducted by a two-tailed equal variance Student’s *t* test. Bars represented the mean  ±  SEM; *P  <  0.05. **c** Statistical analysis of the ANT1 signals between the MPTP-treated group and the control. The analyses were conducted by a two-tailed equal variance Student’s *t *test. Bars represented the mean  ±  SEM; *P  <  0.05. **d** Representative images of ANT1 expression detected by IHC in the different specialized structures of mouse brains. The representative micrographs were taken at magnification of 400. Dark brown signal corresponded to ANT1 stained by anti-ANT1 monoclonal antibody

To characterize the relationship between ANT1 and PD, we analyzed the abundance of soluble ANT1 in the different specialized structures of the mouse brains including hippocampus, cortex, striatum, cerebellum, midbrain and brain stem. Figure 2A shows that soluble ANT1 is expressed at variable levels in the different brain structures. To examine the association of the soluble ANT1 with PD, the relative abundance of soluble ANT1 was assessed using Image J and SPSS 19 as shown in Fig. 2B. Contrast analyses revealed that a significant reduction in soluble ANT1 was found in midbrain (Control: 1.377 ± 0.105; PD: 1.087 ± 0.128; *P * =  0.039), striatum (Control: 1.546  ±  0.025; PD: 0.535  ±  0.024; *P * <  0.001), hippocampus (Control: 1.566  ±  0.136; PD: 0.703  ±  0.040; *P * <  0.001), cerebellum (Control: 1.055  ±  0.053; PD: 0.647  ±  0.034; *P * <  0.001) and brain stem (Control: 1.397  ±  0.166; PD: 0.945  ±  0.069; *P * =  0.012) in the MPTP-induced group, with the exception of cortex. No significance in soluble ANT1 abundance was found in cortex (Control: 1.439  ±  0.150; PD: 1.292  ±  0.0327; *P * =  0.173) between the MPTP-induced group and their controls. This suggests that soluble ANT1 was down-regulated in PD conditions. Thus, the down-regulated soluble ANT1 is found to be associated with PD.

### ANT1 deposition was correlated with PD pathogenesis

To further investigate whether ANT1 was associated with PD, a tissue-based IHC was performed using the monoclonal anti-ANT1 antibody as the probe. The dark brown spot was recognized as positive. Figure 2D demonstrates an apparent expression and distribution of ANT1 in the mouse brains. Strong immunoactivity against anti-ANT1 was detected. The statistic analysis on the ratio of IOD (integrated optical density) to area indicated that there was no significant difference in mouse brains between the MPTP-induced group compared to the control with the exception of brainstem, although there was a decline tendency to ANT1 expression in most special structures. A significant decrease in ANT1 expression was only detected in brainstem (Fig. 2C). That results were not consistent with the ones obtained by Western blot analysis. Based on the contradictory results obtained by IHC and Western blot, we suspected that ANT1 was deposited in mouse brains in insoluble form, and the insoluble ANT1 may damage the neuronal cells to be involved in the pathogenesis of PD.

### Co-aggregation of ANT1 with α-synuclein was found to be associated with PD pathogenesis

**Fig. 3:**
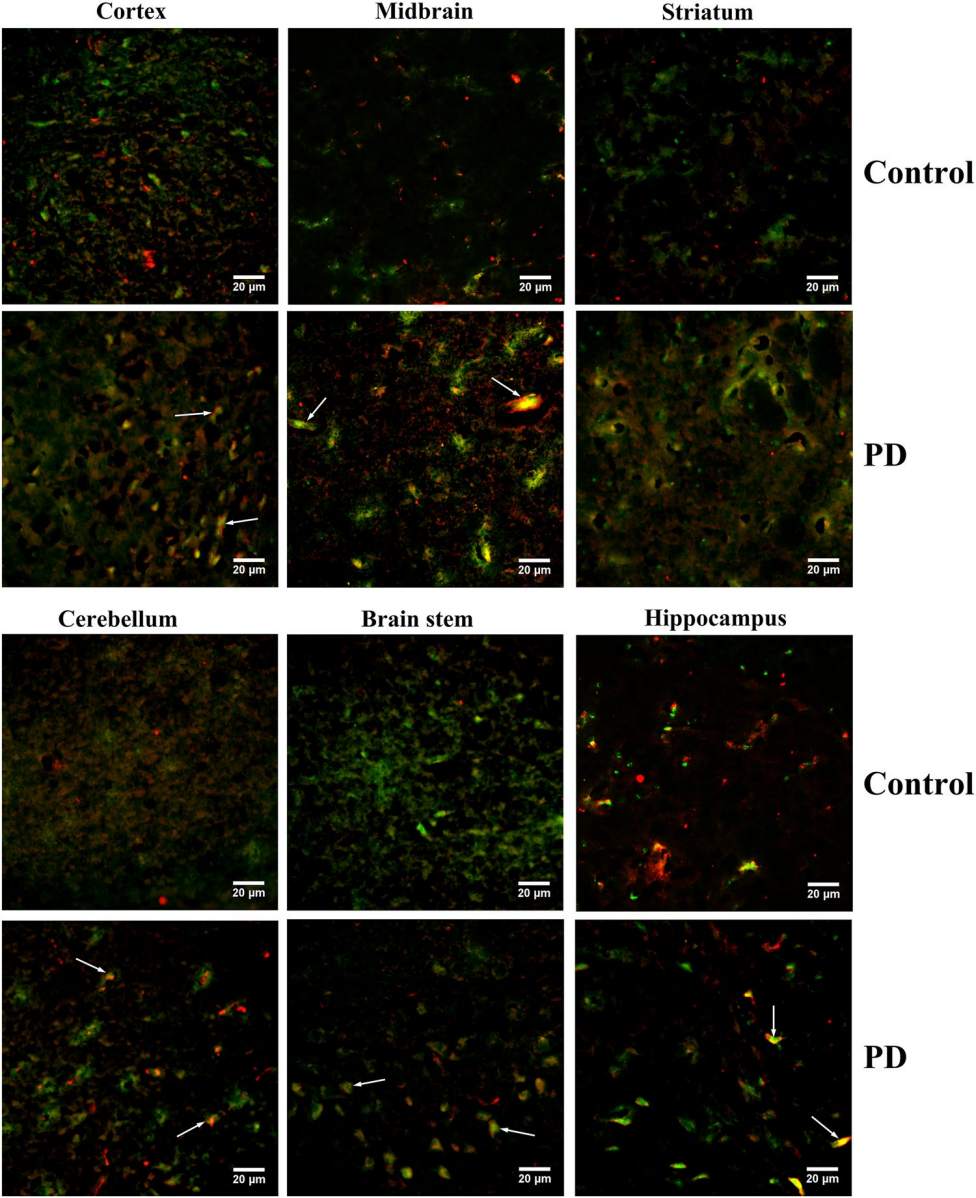
Dual immunofluorescence analysis on the co-aggregation of α-synuclein with ANT1 in the mouse brains. Representative micrographs of the dual immunofluorescence analysis were taken at magnification of 400. Green signal corresponded to ANT1 stained by the monoclonal anti-ANT1 antibody; Red signal corresponded to the α-synuclein detected by the monoclonal anti-α-synuclein antibody; Blue signal corresponded to DAPI-stained nuclei; Co-expression of ANT1 with α-synuclein was shown as the merged images of green and red. The co-aggregation of ANT1 with α-synuclein was indicated by arrows

The aggregation of abnormally folded proteins has been characterized as a common cause of PD, and α-synuclein has been characterized as a major component in protein aggregates. Since the important roles of α-synuclein in PD pathogenesis, an association of ANT1 with α-synuclein aggregation was investigated in the mouse brains using dual immunofluorescence to further reveal the pathogenesis associated with ANT1. We analyzed the co-expression of α-synuclein and ANT1 in the different structures of mouse brains as shown in Fig. 3. Subsequently, an apparent distribution and expression of α-synuclein and ANT1 was found in the above structures of mouse brains. And an obvious co-aggregation of ANT1 with α-synuclein appeared in the structures of cortex, midbrain, striatum, brainstem and hippocampus only in the MPTP-treated group, with the exception of cerebellum. There was no any obvious co-aggregation of ANT1 with α-synuclein in cerebellum whatever in the MPTP-treated group or their controls. Interestingly, we found that ANT1 was located in the center of the protein accumulation, whereas α-synuclein wound around the centric ANT1 in the protein aggregates appeared in the MPTP-treated mouse brains (Fig. 3).

As noted above, although ANT1 was down-regulated in the MPTP-treated group, the protein aggregates formed by ANT1 and α-synuclein was found only in the mouse brains of MPTP-treated group. Thus, co-aggregation of ANT1 with α-synuclein is highly suspected to be associated with PD pathogenesis.

### ANT1 was confirmed to form protein aggregates with α-synuclein at cellular level

In this study, ANT1 was demonstrated to be involved in the pathogenesis of PD via forming protein aggregates with α-synuclein. To further confirm the association of ANT1 with α-synuclein, we aimed to detect this finding at a cellular level. Thereby, neuroblastoma SH-SY5Y cells treated with MPP^+^ was used as a PD-like cell model in this study.

**Fig. 4:**
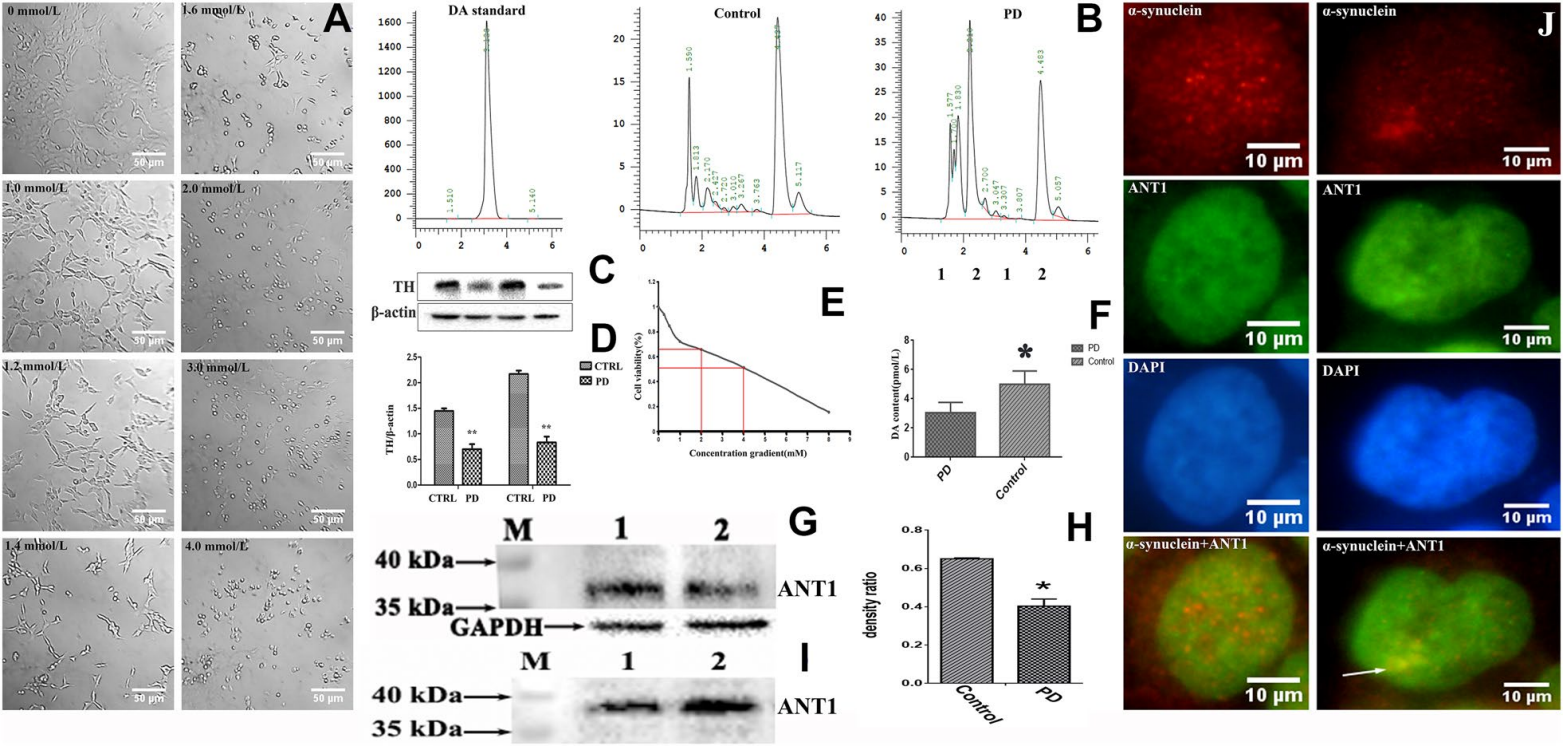
Co-aggregation analysis of ANT1 with α-synuclein at cellular level. Control/CTRL or 1, untreated SH-SY5Y cells; PD or 2, MPP^+^-treated SH-SY5Y cells. **a** Morphological effects of MPP^+^ on the viability of neuroblastoma SH-SY5Y cells. Cells were incubated with different concentrations of MPP^+^ ranging from 0 to 4 mmol/L for another 24 h. The morphology of the SH-SY5Ycells were photographed under a bright field optical microscope at the magnification of 200. Scale bar represented 50 μm. **b** Representative HPLC analysis on cytosolic DA and its standard. **c** Statistical analysis of cytosolic DA concentration from HPLC analysis between the MPP^+^-treated and untreated SH-SY5Y cells. The statistic analyses were conducted by a two-tailed equal variance Student’s *t*-test; bars represented the mean  ±  SEM; *P  <  0.05. **d** Effects of MPP^+^ on the viability of SH-SY5Y cells. Cells were exposed to MPP^+^ with different concentrations ranging from 0 to 8 mmol/L for another 24 h. Cell viability was assessed by MTT assay and expressed as a percentage. **e** Expression analysis on intracellular TH in SH-SY5Y cells detected by Western blot analysis. Cells lysates containing 60 μg soluble proteins were subjected to Western blot analysis coupled with 12% SDS-PAGE separation. The membrane was probed with monoclonal anti-TH antibody and visualized using a ECL kit. **f** Densitometric analysis of intracellular TH normalized to β-actin in SH-SY5Y cells. The statistical analyses were performed by a two-tailed equal variance Student’s t test; Bars represented the mean  ±  SEM; *P  <  0.05. **g** Expression analysis on intracellular ANT1 in SH-SY5Y cells. Eighty microgram of total protein was applied. The membrane was probed with monoclonal anti-ANT1 antibody, and the visualization of bands was performed using ECL. M, Page Ruler prestained protein ladder (Fermentas). **h** Densitometric analysis of soluble ANT1 normalized to GAPDH in SH-SY5Y cells. The statistical analyses were performed by a two-tailed equal variance Student’s *t *test; Bars represented the mean  ±  SEM; *P  <  0.05. **i** Dual immunofluorescence analysis on the co-aggregation of α-synuclein with ANT1 in SH-SY5Y cells. Visualization was performed using an inverted fluorescence microscopy at magnification of 400. Red signal corresponded to the α-synuclein; Green signal corresponded to ANT1; Blue signal corresponded to DAPI-stained nuclei; Co-expression of ANT1 with α-synuclein was shown as the merged images of green and red. The co-aggregation region was indicated by arrows

The optimum working concentration of MPP^+^ on SH-SY5Y cells was evaluated by observing the cellular morphology (Fig. 4A) and detecting the cell viability (Fig. 4D). The viability of SH-SY5Y cells were evaluated using the conventional MTT (3-[4,5-dimethylthiazol-2-yl]-2,5-diphenyltetrazolium bromide) assay. Figure 4D displays that MPP^+^ treatment remarkably reduces the viability of SH-SY5Y cells in a dose-dependent manner. As depicted in Fig. 4A, MPP^+^-treated SH-SY5Y cells became round in shape as the increase of MPP^+^ concentration, whereas the untreated SH-SY5Y cells remained anchorage-dependent growth and the shape of regular polygon. Based on the MTT assays and morphological observation on the MPP^+^-treated cells, 1.2 mmol/L MPP^+^ was determined to induce SH-SY5Y cells to produce parkinsonism symptoms. The MPP^+^-treated cell model was verified by detecting the concentration of cytosolic DA using HPLC and intracellular TH using Western blot analysis. HPLC analysis revealed a dramatic reduction in cytosolic DA in the MPP^+^-treated cells compared to the control cells as shown in Fig. 4B, 4C. TH expression was examined by Western blot analysis as shown in Fig. 4E, and the compare analysis on TH expression revealed a significant decrease in the MPP^+^-treated cells relative to the untreated cells (Fig. 4F). Thereby, the reduction in DA content and TH abundance illustrated that the MPP^+^-treated cells generated a PD-like variation.

Consistent with the results in animal, the low level of soluble ANT1 was found in the MPP^+^-treated cells (Fig. 4G, H). And Fig. 4H demonstrates a statically significant reduction in soluble ANT1 in the MPP^+^-treated cells relative to their control cells. In order to further investigate the possible cause of the soluble ANT1 decrease, we analyzed the ANT1 abundance in the precipitation of cellular extraction. The precipitates were dissolved according to ratio of weight to buffer after the extra buffer was removed by filter paper, then were separated by SDS-PAGE electrophoresis followed by Western blot analysis. Figure 4I illustrates that the clear ANT1 bands are visible in the insoluble protein extraction, and significantly higher in the MPP^+^-treated cells than that in control cells. These evidences demonstrated that the decrease of soluble ANT1 possibly resulted from ANT1 deposition in the forms of insoluble precipitations at cellular level. Thus, it was consistent with the observation by IHC in the different structures of mouse brains.

To further examine whether ANT1 co-aggregated with α-synuclein at cellular level, a co-expression analysis on ANT1 and α-synuclein in SH-SY5Y cells was performed using double-immunofluorescence as shown in Fig. 4J. The monoclonal anti-ANT1 antibody specifically recognizes ANT1 in green, and the monoclonal anti-α-synuclein antibody specifically interacts with α-synuclein in red. The co-localization of ANT1 with α-synuclein was analyzed using the merged images. Figure 4J shows that SH-SY5Y cells display strong immunoreactivities against anti-α-synuclein antibody, and many brightly red dot-like structures are evident in the cells. Additionally, a large area with brightly red signals was found in the MPP^+^-treated cells, but not in the untreated cells. Unlike the regular and clear dot-like structures in the SH-SY5Y cells, this irregular large area with brightly red signals was highly suspected to result from protein accumulation. ANT1 indicated in brightly green was expressed in scattered dots in SH-SY5Y cells, and similar irregular large area with brightly green signals was found only in the MPP^+^-treated cells relative to the untreated cells (Fig. 4J). Co-localization analysis indicated that ANT1 and α-synuclein existed independently in SH-SY5Y cells normally, and no co-expression was detected between the ANT1 and α-synuclein in the untreated cells. However, the co-localization of ANT1 and α-synuclein was detected in the MPP^+^-treated cells, and only in the region with large area of protein aggregates as shown by the arrow in Fig. 4J. Therefore, the above evidences illustrate that ANT1 can interact with α-synuclein to promote the formation of protein aggregates only in the MPP^+^-treated SH-SY5Y cells. This is consistent with conclusions drawn at the animal level.

### ***ANT1 supplement ameliorated MPP***^+^***-induced cytotoxicity in neuroblastoma cells***

**Fig. 5:**
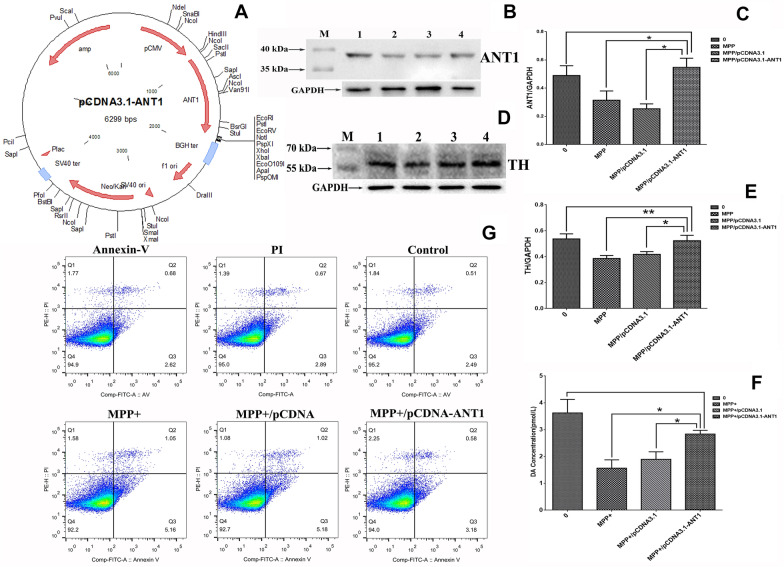
Effect of ANT1 supplement on MPP^+^-treated neuroblastoma SH-SY5Y cells. **a** The map of pcDNA3.1-ANT1 which was transfected into the MPP^+^-treated neuroblastoma SH-SY5Y cells and overexpressed ANT1 protein. **b** Analysis of ANT1 abundance in various cell models detected by Western blot. Cells lysates containing 80 μg soluble proteins were subjected to Western blot analysis coupled with 15% SDS-PAGE separation. The membrane was probed with monoclonal anti-ANT1 antibody and visualized using a ECL kit. M, Page Ruler prestained protein ladder (Fermentas). **c** Densitometric analysis of ANT1 normalized to the level of GAPDH. The analyses were conducted by a two-tailed equal variance Student’s *t *test. Bars represented the mean  ±  SEM; *P  <  0.05. **d** Analysis of TH abundance in various cell models detected by Western blot analysis. Cells lysates containing 60 μg soluble proteins were subjected to Western blot analysis coupled with 10% SDS-PAGE separation. The membrane was probed with monoclonal anti-TH antibody and visualized using a ECL kit. M, Page Ruler prestained protein ladder (Fermentas). **e** Densitometric analysis of TH normalized to the level of GAPDH. The analyses were conducted by a two-tailed equal variance Student’s *t *test. Bars represented the mean  ±  SEM; *P  <  0.05; **P  <  0.01. **f** Densitometric analysis of DA levels detected by HPLC. **g** Apoptosis analysis of neuroblastoma cells using AnnexinV-FITC/PI staining and flow cytometry

To further investigate the association of ANT1 with PD, we introduced the plasmid of pcDNA3.1-ANT1 (Fig. 5A) into the MPP^+^-treated neuroblastoma SH-SY5Y cells for over-expressing ANT1 protein. The transfection efficiency was confirmed using Western blot probed with monoclonal anti-ANT1 antibody. Figure 5B, C show that the transfected cells with pcDNA3.1-ANT1 dramatically raise the ANT1 abundance in the MPP^+^-treated SH-SY5Y cells. In this study, the untreated cells, and MPP^+^-treated cells which were transfected with pcDNA3.1 were used as the controls.

To examine whether the ANT1 supplement can ameliorate cytotoxicity caused by MPP^+^ in neuroblastoma cells, we tested the typical parameters of PD including TH and DA as shown in Fig. 5D–F. TH abundance analyzed by Western blot and DA level examined by HPLC didn’t show a significant difference between the MPP^+^-treated and untreated SH-SY5Y cells. Meanwhile, the apoptosis of neuroblastoma cells was analyzed using AnnexinV-FITC/PI staining and flow cytometry as depicted in Fig. 5G. The percentages of early apoptotic cells were decreased from 5.18 to 3.18%, and late apoptotic cells were dropped from 1.02 to 0.58% compared with the MPP^+^-treated SH-SY5Y cells which was transfected with pcDNA3.1. Thereby, ANT1 supplement contributes to the neuroprotective effects in MPP^+^-treated cell models. Thus, it further demonstrated that ANT1 supplement attenuated MPP^+^-induced cytotoxicity in SH-SY5Y cell models.

### In vitro* evidences confirmed the interaction of ANT1 with α-synuclein*

**Fig. 6:**
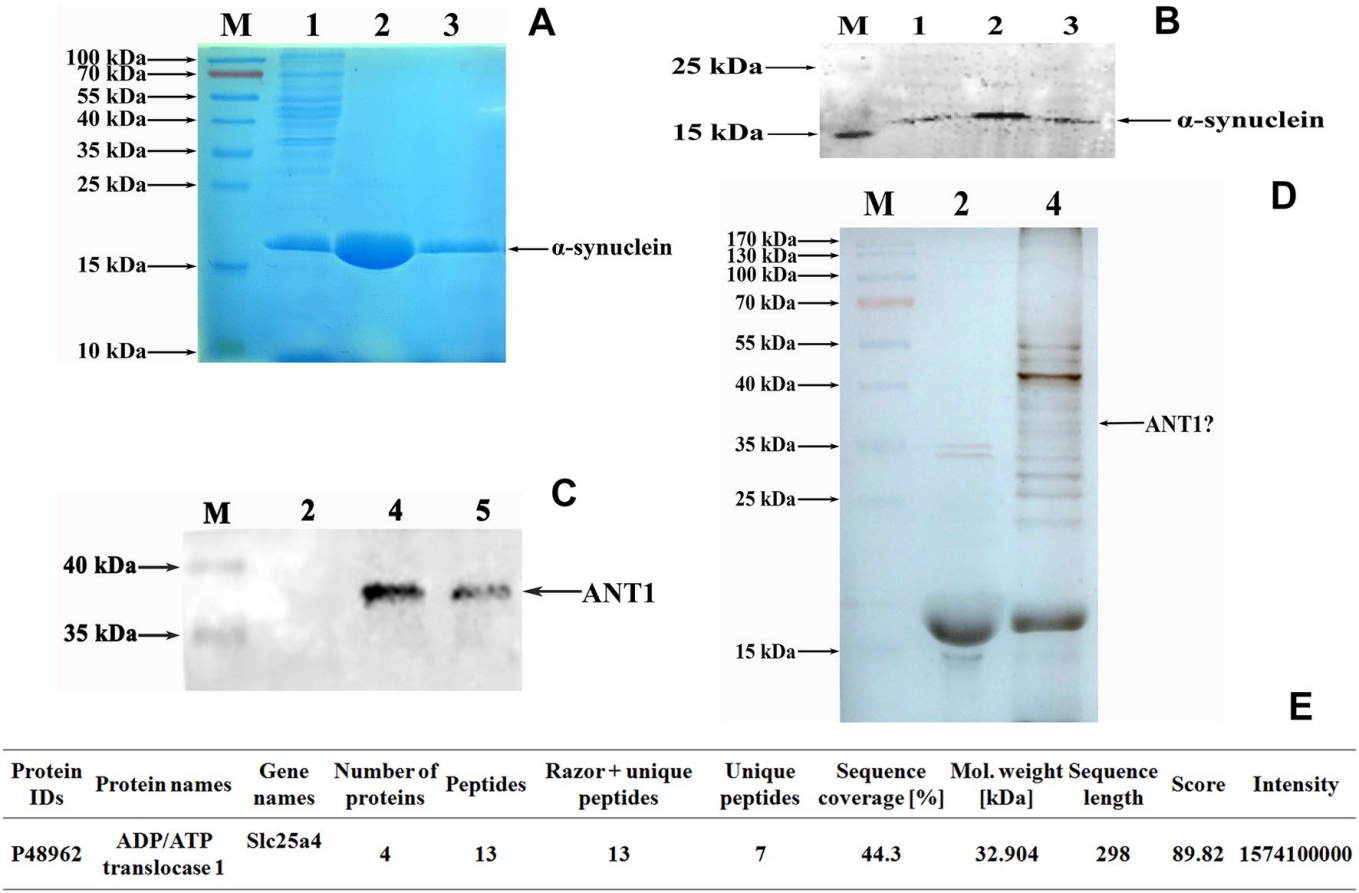
Confirmation of ANT1 interaction with α-synuclein in vitro. M, Page Ruler prestained protein ladder (Fermentas); 1, cell lysate of *E.coli* containing over-expressed α-synuclein; 2, eluent fraction-1 from Ni-NTA affinity chromatography (purified α-synuclein); 3, eluent fraction-2 from Ni-NTA affinity chromatography (purified α-synuclein); 4, eluent fraction from pull-down assay (interaction proteins); 5, cell lysates of mouse brains. **a** SDS-PAGE analysis of α-synuclein expressed in *E.coli*. The gel was stained with Coomassie blue. **b** Western blot analysis of the purified α-synuclein using monoclonal anti-6XHis antibody as the probe. The soluble α-synuclein was purified by Ni-NTA affinity chromatograph. **c** SDS-PAGE analysis of interaction proteins with α-synuclein. The interaction proteins were pulled down using Ni-NTA magnetic agarose beads as the medium and the purified α-synuclein as the bite. The gel was visualized by silver staining. **d** Western blot analysis of interaction proteins with α- synuclein against anti-ANT1 monoclonal antibody. **e** Profiles of the identified ANT1 in Mus musculus by LC-MS/MS

As noted, ANT1 specifically interacted with α-synuclein to form protein aggregates in PD-like models at the animal and cellular level. In order to clarify the association of ANT1 with PD, an in vitro protein interaction assay was performed using a pull-down technique. In the protein interaction assay applied in this study, α-synuclein was used as a bait to fish its interaction partners. α-synuclein was prepared by over-expression in *E. coli* using IPTG as an inducer, and purified by Ni–NTA gel affinity chromatography. The purified α-synuclein was verified by SDS-PAGE gel stained with Coomassie blue (Fig. 6A) and Western-blot using anti-6XHis monoclonal antibody as the probe (Fig. 6B). Thus, Fig. 6 shows that the purified α-synuclein is successfully obtained and available for a further investigation.

A pull-down assay was conducted for fishing the interaction partners of α-synuclein using Ni-NTA magnetic agarose beads as the medium, and α-synuclein as the bait. Consequently, the interaction analysis revealed that there were bands between 35 and 40 kDa which were consistent with the size of ANT1 as shown in Fig. 6C. In addition, LC-MS/MS analysis was applied to identify the interaction partners of α-synuclein as well, and the mass spectra displayed that ANT1 was one of interaction partners of α-synuclein (Fig. 6E). To confirm the results of LC-MS/MS analysis, Western-blot analysis was conducted using anti-ANT1 monoclonal antibody as the probe. Figure 6D shows a strong immunoreactivity against anti-ANT1 antibody at the molecular weight of 35 to 40 kDa in the samples pulled down by α-synuclein. So the Western-Blotting analysis further confirmed that ANT1 was the interaction partner of α-synuclein.

Based on the evidences obtained in this study, we proposed a hypothesis that ANT1 co-aggregated with α-synuclein to form insoluble protein inclusions which play a role in the pathogenesis of PD.

## Discussion

ANT1 is a nuclear-encoded polypeptide with a mass of 33.064 kDa and 298-amino-acid based on its sequence in homo sapient which was obtained from the public online database: https://www.ncbi.nlm.nih.gov/protein/NP_001142.2. ANT1 is highly conserved in mammalian species. For example, the homology in the coding sequences of ANT1 extends remarkable to 96% between homo sapient and rattus norvegicus, and to 95% between homo sapient and mus musculus.

**Fig. 7:**
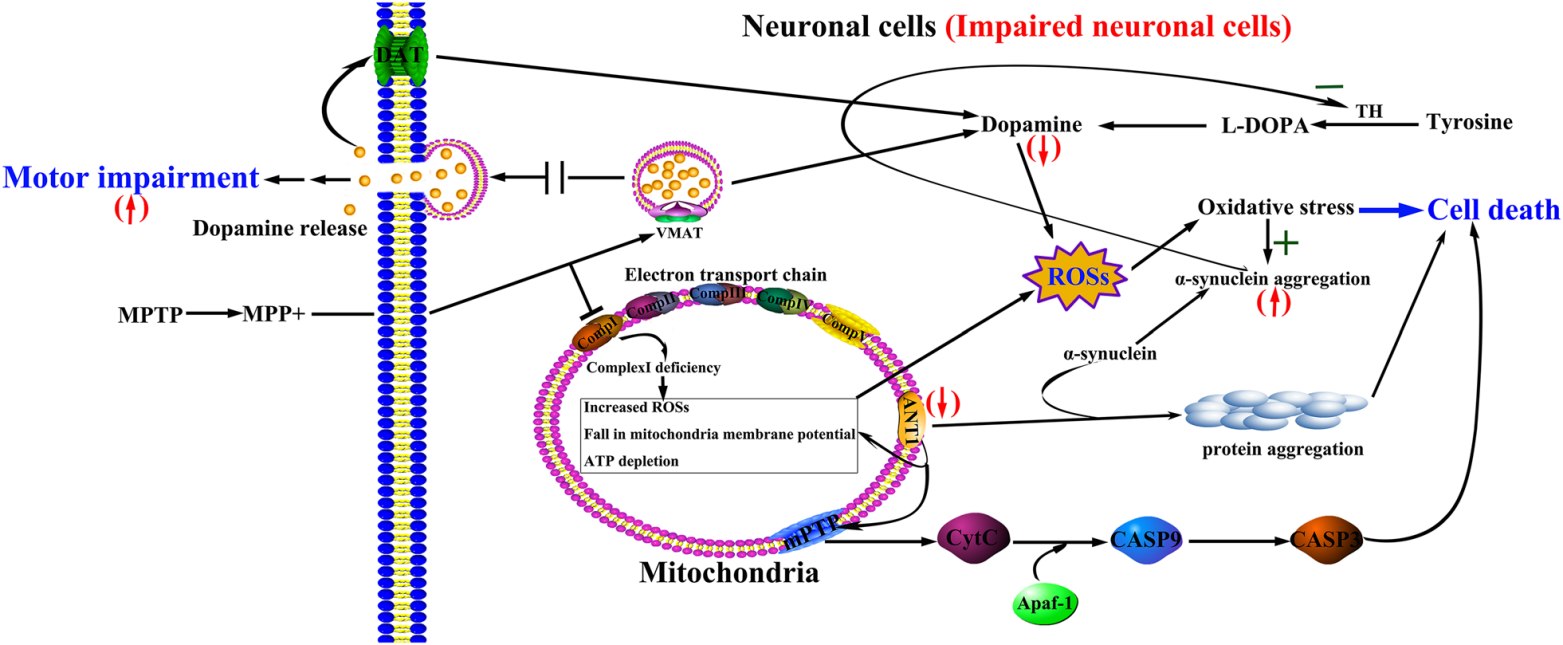
Hypothetical conclusive mechanism of neurodegenerative disorder associated with ANT1. The pathway was summarized according to the pathway shown in KEGG website (https://www.kegg.jp/kegg-bin/highlight_pathway?scale=1.0&map=hsa05012&keyword=MPTP). Comp, complex; VMAT, vesicular monoamine transporter; DAT, dopamine transporter; TH, tyrosine hydroxylase; mPTP is composed of ANT1, cytosolic benzodiazepine receptor, Bax, and CypD. Red arrows displayed the detected changes occurred in impaired neuronal cells in this study. “ + ” indicated the promoted processes, “−” indicated the inhibited processes

In this study, ANT1 was found to be associated with PD. As reported, the impairment of ANT1 is proposed to contribute to the pathogenesis of mitochondrial myopathy and hypertrophic cardiomyopathy [26]. A deficit of mitochondrial ANT1 is implicated in cellular dysfunction in type 2 diabetes and obesity by raising adenosine and reactive oxygen species [14]. The dysfunction of ANT1 results in an increasing reactive oxidative species (ROSs), fall in mitochondiral potential, and ATP depletion (Fig. 7). It is reported that ANT1 deficits associated with increased mitochondrial ROSs [13]. Excessive generation of ROSs and long-term exposure to ROSs may lead to age-dependent degenerative diseases such as PD. ROSs are involved in the oxidative damages to biomacromolecules such as proteins, nucleic acids and lipids, resulting in alteration of intrinsic membrane properties like fluidity, mitochondrial dysfunction, dopaminergic neuron degeneration, impaired cellular functions and ultimately progressing to PD [27]. In this study, in order to evaluate the association of ANT1 with ROSs, we detected intracellular ROSs in neuroblastoma SH-SY5Y cells. Compared to the untreated SH-SY5Y cells (245.67  ±  8.02), the increment rate of ROSs level in the MPP^+^-treated SH-SY5Y cells (293.00  ±  22.52) was as high as 19.27% (*P * =  0.027). Thus, ROSs level was significantly increased in MPP^+^-treated neuroblastoma cells. Based on this finding, we highly suspected that down-regulated ANT1 resulted in the dysfunctional transport of ATP and ADP across the mitochondrial membrane, and led to the abnormal production and accumulation of ROSs, then participated in the pathogenesis of PD. In addition, oxidative stress is thought to promote the accumulation of α-synuclein, a hallmarker of PD, further to promote the formation of Lewy bodies in brain [27–29]. So ANT1 potentially is a defective factor to cause PD.

Although ANT1 has been shown to be cytotoxic in several cell types and induce cell death in cardiomyocytes and Hela cells [12, 18], there is a controversial study which indicate that ANT1 overexpression protects hypoxic cardiomyocytes against oxidative stress and increases cell survival [30]. Till now, the physiological roles of ANT1 are still under debate. Our study clearly showed that ANT1 supplement played a role in protecting neuroblastoma cells against MPP^+^-induced cytotoxicity and rescuing cells from the PD-like injury.

In this study, we illustrated that dysfunction of ANT1 led to the formation of the α-synuclein-containing protein aggregates. Based on the evidences obtained in this study, we proposed a hypothesis that the down-regulated ANT1 was the source of the increased ROSs, and the dysfunctional ANT1 initiated the formation of the protein accumulation which was associated with α-synuclein. The increased ROSs may attack α-synuclein to promote the co-aggregation of α-synuclein with ANT1 resulting in cell death of neurons as shown in Fig. 7. Furthermore, the coagulation of ANT1 with α-synuclein possibly resulted in the decrease of ANT1 abundance or undetectable ANT1 in protein aggregation. Thereby, the detectable ANT1 was down-regulated in MPTP/MPP^+^-treated models compared to their controls.

In addition, the importance of ANT1 also lies in its involvement in the formation of pro-apoptotic mPTP (mitochondrial permeability transition pore) as a major component, which is located in the inner membrane of mitochondria. ANT1 interacts with several proteins containing cytosolic benzodiazepine receptor, porin/voltage-dependent anion channel, Bax and matrix cyclophilin-D to form mPTP. In this study, we found a dramatic decrease in ANT1 in MPTP/MPP^+^-treated models. Then, ANT1 impairment possibly results in mPTP open. Theoretically, mPTP open allows an increase in the permeability of molecules with less than 1500 Daltons in mass across the inner membranes of the mitochondria, thus results in mitochondrial swelling [12]. mPTP open causes CytC (cytochrome C) to be released outside mitochondria, then a cascade of cell apoptosis program, intermediated by CytC, Apaf-1 (apoptotic protease activating factor-1), caspase-3 and caspase-9, is triggered in neuronal cells, resulting in the pathogenesis of PD (Fig. 7).

Based on the obtained evidences in this study, an implication of ANT1 for PD pathogenesis is proposed. As a key functional component in the inner membrane of mitochondria, ANT1 is involved in the exchange of cytosolic ADP and mitochondrial ATP, and plays a crucial role in maintaining the mitochondrial function, whereas mitochondrial dysfunction plays a critical role in the aged disease such as PD. In this study, we detected a down-regulated DA and ANT1, and up-regulated α-synuclein aggregation, co-aggregation of ANT1 with α-synuclein, and motor impairment in the MPTP-treated group (Fig. 7). The protein aggregates formed by ANT1 and α-synuclein may damage the neuronal cells. Additionally, insufficient ANT1 cannot maintain its activity in mPTP construction and finally cause mPTP open and initiate a cascade of cell apoptosis program. Thus, in view of the important roles of ANT1 in energy metabolism and its involvement in mPTP construction, ANT1 is highly suspected to have an important implication for PD pathogenesis as a defective factor of PD.

## Conclusions

As noted, we reported that the down-regulated ANT1 and suspicious ANT1 accumulation were associated with PD pathogenesis via forming the co-aggregation with α-synuclein, and ANT1 supplement attenuated MPP^+^-induced cytotoxicity in SH-SY5Y cell models. Despite the limitations of this study, this is the first report to reveal ANT1 as potential etiology of PD. This investigation provides key information necessary for designing prospective studies to evaluate ANT1 in the etiology of PD, thus may potentially provide valuable information on developing potential drug targets in PD treatment or reliable biomarkers in PD prognostication.

## Data Availability

The datasets used and/or analysed during the current study are available from the corresponding author on reasonable request.
